# Clinical Manifestations and Characterization of COVID-19 in Liver Transplant Recipients: A Systematic Review of Case Reports and Case Series

**DOI:** 10.4314/ejhs.v31i2.26

**Published:** 2021-03

**Authors:** Pirouz Samidoust, Hamed Nikoupour, Hossein Hemmati, Aryan Samidoust

**Affiliations:** 1 Razi Clinical Research Development Unit, Razi Hospital, Guilan University of Medical Sciences, Rasht, Iran; 2 Shiraz Transplant Center, Abu Ali Sina Hospital, Shiraz University of Medical Sciences, Shiraz, Iran

**Keywords:** SARS-CoV-2, COVID-19, Liver transplantation, Systematic review

## Abstract

**Background:**

This systematic review is conducted to explore available information on clinical presentations, laboratory finding and outcomes of SARS-COV-2 in liver transplant patients.

**Methods:**

We searched four databases for relevant terms related to COVID-19 and liver transplantation and collected both case reports and case series on liver transplantation published up to the end of September 2020.

**Results:**

After initial screening of irrelevant articles, 25 studies were included and analyzed in this review. Among the 59 patients included, 78.3% were over 50 years old, and 71.6% were males. The majority of patients (93.3%) were hospitalized. The most common presenting symptoms were fever (72.9%) followed by dyspnea and cough (54.2%). The majority of patients revealed a high level of CRP (64.3%). Moreover, high level ALT, AST and ALP were reported in 64.3, 37.5, 30.5 and 22.2% of patients. A total, 9(15.3%), of cases died as a result of complications of COVID-19. Chest radiographs were reported in 72.9%(43/59) of cases that 94% demonstrated radiologic evidence of abnormality.

**Conclusion:**

The results demonstrated that the most prevalent symptoms and signs were fever, dyspnea and cough. Moreover, most patients were males and hospitalized. The rate of mortality and high level of CRP, ALT/AST and ALP is similar within the non-immune suppressed and general population. However, early detection of high level of serum CRP, ALT/AST and ALP combined with a clinical COVID-19 symptom and finding of CT scan may be used as an index for the presence and severity of the disease.

## Introduction

In late December 2019, several very progressive pneumonia-like respiratory syndromes called COVID-19 (coronavirus 2019 disease), first occurred in Wuhan, Hubei Province, China (1–3). Severe acute respiratory syndrome coronavirus 2 (SARS-COV-2) is a zoonotic virus belonging to the Coronaviridae family, single-stranded, enveloped, non-segmented, positive-sense RNA genomes cause symptoms ranging from those similar to the common cold to interstitial pneumonia with acute respiratory distress syndrome (ARDS), with a case mortality percentage close to 8–10% (4–6). The primary transmission route of COVID-19 is through man-to-man via respirational droplets generated by breathing, sneezing, coughing from patients with pneumonia or asymptomatic patients, but the virus can also be transmitted through the fecal-oral route (7,8). Since the beginning of the epidemic, it has increasingly been evidenced that older adults and patients with underlying diseases, including transplant recipients, have a greater risk for the development of life-threatening and lethal respirational illnesses (9,10). However, solid-organ transplant (SOT) receivers, like liver transplants, expectedly develop elevated levels of severe respiratory tract infections and fatality owing to the declined immunity caused by the use of immunosuppressive medications like steroids after organ transplantation (11,12). There are currently limited data on the clinical course, the optimal therapeutic approach, and the outcomes of COVID-19 in the milieu of solid organ transplantation, particularly liver transplant receivers, and the evidence is limited to case reports and small case series (1,13–15). In the European cohort of liver transplant patients infected with COVID-19, Becchetti et al. reported during hospital stay, mortality percentages were 12% and 17%, respectively (16). Another study from Iran indicated that the use of the immunosuppressive drugs not only had no increasing effect on death rates, but also positively affected the reduction of the disease intensity and clinical duration (17).

Since limited information is available about the COVID-19 infection in liver transplant receivers, particularly information regarding the management of immunosuppressant's and fatality rates, this review aimed to conduct a comprehensive systematic review of the available data about clinical presentations, laboratory changes and outcomes of SARSCOV-2 in liver transplant recipients.

## Methods

**Study design and search strategies**: This present review conforms to the “Preferred Reporting Items for Systematic Reviews and Meta-Analyses” (PRISMA) statement. We carried out comprehensive systematic searches of the literature review of Web of Science, PubMed, Embase and Scopus. Search criteria included case reports and case series studies of relevant terms related to COVID-19 and SARS-CoV-2 in liver transplant patients published up to end of September, 2020 in languages other than English.

The search strategy for our review was based on the following keywords: “2019 novel coronavirus” OR “severe acute respiratory syndrome coronavirus 2” OR “SARS-CoV-2 infection” OR “2019- nCoV infection” OR “novel coronavirus” OR “coronavirus disease 2019” OR “COVID-19” AND “Liver” AND “transplant” OR “graft”.

**Study selection**: All studies met the following inclusion criteria: prospective or retrospective descriptive case reports and case series of liver transplants patients infected with COVID-19 which confirmed and diagnosed according to the criteria recommended by WHO. In addition, articles had to include diagnostic methods, clinical symptoms, laboratory or radiological findings, and outcomes. Articles describing other tissue transplantation as well as guidelines, unavailable full texts, duplicate publications without peer review processes and lack of sufficient data were excluded.

In the first stage, two reviewers (PS, AS) independently screened the title and abstracts of all eligible studies. Second, the full text of those abstracts which met the inclusion criteria was considered and independently reviewed by the same authors and disagreements were resolved between review authors (PS, AS and HN).

**Data extraction**: Two authors (PS, HN) independently extracted the data from the eligible studies using a pre-defined data extraction sheet. The following data such as patient demographics (e.g., age, sex), radiological and laboratory data, comorbidities, history of the transplanted organ, COVID-19 clinical manifestations (Signs, Symptoms), RT-PCR tests and patients' outcome were extracted. In the case of inconsistencies, the researchers discussed and resolved between review authors to reach consensus (PS, AS).

**Statistical analysis**: The statistical analysis was carried out using SPSS version 16.0 (SPSS Inc). Continuous variables were assessed as mean ± standard deviation, and categorical variables were reported as counts and percentages.

## Results

**Figure 1:**
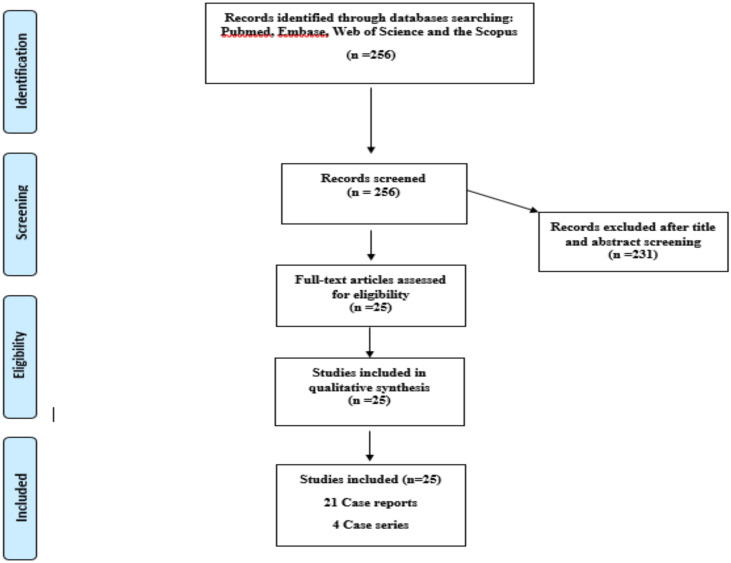
Flow chart of study selection for inclusion in the systematic review

The initial search revealed 256 relevant articles. After initial screening of titles and abstracts, duplicate citations and irrelevant articles were excluded by title and abstract assessment. In the secondary evaluation, 25 studies were included and analyzed in this review (18–42). Figure 1 depicts the flow diagram of the details of the literature search based on the PRISMA statement, as well as the selection criteria for the articles.

Screening the inclusion criteria to the full-text articles, 21 case reports and 4 case series from 8 countries were recognized with 59 unique cases of COVID-19 (Table 1). Studies included reports from the United States, China, Italy, Iran, Spain, France, Brazil, and Denmark.

**Table 1:** Characteristics of the included articles

	Authors	Country	Published time	Type of study	Age	Male/ Female	No. of patient (s)	Diagnostic methods	Outcome
1.	Bin et al	China	2020	Case report	50	M	1	RT-PCR/CT-scan	Recovered
2.	Barros, Machado etal	Brazil	2020	Case report	69	M	1	RT-PCR/CT-scan	Recovered
3.	Donate et al	Italy	2020	Case series	63	6M,2F	8	RT-PCR/CT-scan	6 Recovered, 2 Hospitalized
4.	Gao et al	China	2020	Case report	48.6	3M	3	RT-PCR/CT-scan	1 Expired, 2 Recovered
5.	Hammami et al	USA	2020	Case report	63	M	1	RT-PCR/CT-scan	Recovered
6.	Kates et al	USA	2020	Case report	67	M	1	RT-PCR	Recovered
7.	Zhong et al	China	2020	Case report	37	M	1	RT-PCR	Recovered
8.	Qin et al	China	2020	Case report	37	M	1	RT-PCR	Recovered
9.	Morand et al	France	2020	Case report	55 months	IF	1	RT-PCR	Recovered
10.	Huang et al	China	2020	Case report	59	M	1	RT-PCR/CT-scan	Expired
11.	Bhoori et al	Italy	2020	Case report	>65y	3M	3	RT-PCR	Expired
12.	Ruiz et al.	Spain	2020	Case series	68	3M,3F	6	RT-PCR/CT-scan	2 Expired, 4 Recovered
13.	Nikoupour et al	Iran	2020	Case report	35	M	1	RT-PCR	Recovered
14.	Niknam et al	Iran	2020	Case series	53	1M, IF	2	RT-PCR/CT-scan	Recovered
15.	Stephen et al	USA	2020	Case report	6 months	F	1	RT-PCR	Recovered
16.	Heinz et al	USA	2020	Case report	6 months	F	1	RT-PCR	Recovered
17.	Nikoupour et al	Iran	2020	Case report	3	M	1	RT-PCR	Expired
18.	Mathiasen et al	Denmark	2020	Case report	58	F	1	RT-PCR	Recovered
19.	Maggi et al	Italy	2020	Case report	65	2M	2	RT-PCR	1 Expired, 1 Recovered
20.	Fung et al	USA	2020	Case report	80	F	1	RT-PCR	hospitalized
21.	Loinaz et al	Spain	2020	Case series	62	5F, 12M	19	RT-PCR	2 Expired, 15 Recovered
22.	Modi et al	USA	2020	Case report	32	M	1	RT-PCR	Recovered
23.	Prieto et al	Spain	2020	Case report	52	M	1	RT-PCR	Recovered
24.	Antony et al	USA	2020	Case report	63	M	1	RT-PCR	Recovered
25.	Song et al	China	2020	Case report	37	M	1	RT-PCR	Recovered

Among the 59 patients included, 78.3% were over 50 years old, and 71.6% were males. The patients' mean and age range were 50.8 (6m-80y). The majority, 68.3%(41/60), of the patients experienced infection 2 years after transplantation, and only 10 patients were diagnosed during the first year of transplant (19,20,26,27,30,34,37–43). The majority of the patients (93.3%) were hospitalized during the course of illness.

The most common presenting symptoms were fever (72.9%), dyspnea and cough (54.2%), followed by myalgia/fatigue, sore throat, headache, diarrhea and chills (Table 2). Additionally, in studies describing C-reactive protein (CRP) values, the majority of the patients revealed a high level of CRP (64.3%). Moreover, high-levels of ALT, AST and ALP were reported in 64.3, 37.5, 30.5 and 22.2% of patients, respectively. We found 3/60 (13.2%) reports on Acute Respiratory Distress Syndrome (ARDS). Mortality outcomes were reported in all of studies. Furthermore, 9(15.3%) of the cases died from complications of COVID-19. Among reported comorbidities such as pulmonary disease, diabetes, hypertension, hepatitis B cirrhosis and cardiovascular disease, hypertension was the most prevalent with 22% (13/59) frequency. Chest radiograph was reported in 72.9% (43/59) of the cases. Of these, 94% demonstrated the radiologic evidence of abnormality. Chest CT scan was frequently reported with ground-glassopacity (GGO) patterns (75%) as well as with bilateral lung involvement (75%).

**Table 2:** Summary of the case report and case series findings

Variables		Number*	Percent
Clinical	Fever	43/59	72.9
manifestations	Cough	32/59	54.2
	Dyspnea	32/59	54.2
	Myalgia/fatigue	12/59	20.3
	Sore throat	5/59	8.5
	Headache	5/59	8.5
	Diarrhea	8/59	13.6
	Rhinorrhea	0/59	0
	Chills	3/59	5.1
	High CRP	18/28	64.3
			Range: 1–102 mg/L
	High AST	12/32	37.5
			Range: 17–770 U/L
	High ALT	11/36	30.5
			Range: 3–333 U/L
	ALP	6/27	22.2
			Range: 41–1140 U/L
CT	Both of GGO and Consolidation	3/12	25
	GGO without Consolidation	9/12	75
	Unilateral	3/12	25
	Bi lateral	9/12	75
Complications	ARDS	3/59	5.1
	Hospitalization	56/59	93.3
Outcomes	Discharged	51/59	85
	Death	9/59	15.3

## Discussion

In this study, we assessed the clinical characteristics and epidemiologic features of case series and case report of liver transplant patients to gain a better understanding of the true risk of this complication caused by SARS CoV-2 in liver transplant recipients. The previous literature review and case series affirm the notion that COVID-19 disease occurs more severely in the receivers of solid organ transplants and are further disposed to hospital stay with greater fatality percentages due to COVID-19 compared to the patients without transplants (44,45). However, there are limited reviews on case reports about SOT patients (6,44).

Here, we summarized the details and characteristics of 59 liver transplant recipients who developed COVID-19, including nine (15.3%) who died from respiratory failure. This trial recorded almost two-thirds of male patients aged around 50.8 years on average. In a recently published meta-analysis, 337 transplantation patients were at the age of 49.9 years on average, and men comprised 70% of patients (10). Nevertheless, the greater percentage of COVID-19 in male transplant receivers could be because men comprised a greater percentage in transplant patients according to the literature (46).

Furthermore, the association between the SOT status and fatality was examined in a descriptive study. The results of this study indicate that the fatality rate among COVID-19 SOT receivers is the same as general population; however, higher rate of respiratory failure was observed in SOT recipients than in the general population (9).

Based on our results, it appears that the timeline of transplantation was not considered as an important role in the development of infection in these cases. Contrary to our results, Rinaldi et al. demonstrated that the timetable of transplantation apparently considerably contributed to the progress of infection, where they reported greater levels of acute respirational failure among patients who experienced transplantation during 1 year from COVID-19 detection compared to lately transplanted receivers (9). Their results suggest that the severity of immunosuppression apparently strongly correlates with the risk of developing a more acute disease.

According to our reviewed articles, hypertension, diabetes, and pulmonary disease were the most common co-morbidities among COVID-19 patients, which were associated with a more severe clinical course (47); this is also mentioned in our findings and is consistent with reports by Fraser et al. and Tahvildari et al. (44,48). In this regard, Fraser et al., in one quantitative analysis on liver transplant recipients reported that ages over 60 years old and diabetes mellitus were significantly associated with fatality (44). Therefore, they recommended that liver transplant patients with common comorbidities would require close monitoring.

According to the review studies, the most prevalent symptoms and signs were fever, dyspnea, cough, and myalgia/fatigue, while diarrhea, headache, sore throat and chills were less common clinical manifestations (9,11,28,41,49). Although transplanted patients may represent with slight or unusual signs and with no fever, doctors must adopt wide differential diagnostic procedures and high suspicion clinically (43). Additionally, the present findings totally correspond to the same clinical profile of the general population with highly widespread indications such as fever, cough, and dyspnea. In line with our findings, a case series study of five transplant patients in China demonstrated that highly widespread indications on admittance were fever, cough, myalgia/fatigue, and sputum production (25).

According to previous reports, gastrointestinal indications were more frequent in liver transplant receivers with COVID-19 (28.6%) than in the general population, being partially comparable to our reports among solid organ transplant cases (44). The most frequent abnormalities were high level of CRP, ALT/AST and ALP. These laboratory abnormalities may indicate the severity of disease and developing complications in LTP (50). The percentages of hepatitis were apparently greater inpatients with acute COVID-19 exhibiting elevated liver enzyme than slight to medium illness (50). Interestingly, patients with high levels of ALT and AST underwent transplantation during two years from COVID-19 detection.

Accordingly, Tahvildari et al., in one meta-analysis, reported that 60, 48 and 28.5% of patients had high levels of CRP, ALT and AST, being partially in agreement with our results (48).

Moreover, in a meta-analysis that systematically reviewed 20 investigations with 3428 COVID-19 cases, elevated serum concentrations of AST, ALT, and total bilirubin were correlated with significantly increased intensity of COVID-19(51). One point to be considered is that close monitoring and management of immunosuppressant treatment in transplant patients affected by COVID-19 are essential. The standard and balanced treatment dosing allows sufficient immune reaction to repress viral loading and prevent transplant refusal (44).

However, antiviral treatment in isolation is not a sufficient therapy and supports the theory that ARDS is the result of the cytokine storm. In this regard, Zhong et al., in a liver-transplanted patient with COVID-19, notices that acute transplant rejection occurred despite retaining sufficient immunosuppression according to lymphocyte subtests. Among 51 patients with reports of radiological results, 94% possessed radiographic or CT results “indicative” of being diagnosed with COVID-19 (28,43).

In conclusion, we investigated and summarized the clinical characteristics, laboratory abnormalities, common comorbidities, and imaging modalities in the liver-transplanted population with COVID-19. The results demonstrated that the most prevalent symptoms and signs were fever, dyspnea, cough, and myalgia/fatigue. Moreover, most patients were males and hospitalized.

The rate of mortality and high level of CRP, ALT/AST and ALP is similar within the non-immune suppressed and general population. However, early detection of high level of serum CRP, ALT/AST and ALP combined with a clinical COVID-19 symptom and CT scan findings may be used as an index for the presence and severity of the disease.
